# Mental healthcare users’ self-reported medication adherence and their perception of the nursing presence of registered nurses in primary healthcare

**DOI:** 10.4102/hsag.v26i0.1618

**Published:** 2021-07-22

**Authors:** Lillian Kalimashe, Emmerentia du Plessis

**Affiliations:** 1 Department of Health, Faculty of Health Sciences, West Rand Health District, Gauteng Department of Health, Johannesburg, South Africa; 2 NuMIQ Research Focus Area, Faculty of Health Science, North-West University, Potchefstroom, South Africa

**Keywords:** mental healthcare users, medication adherence, nursing presence, registered nurses, primary healthcare

## Abstract

**Background:**

Medication adherence remains a challenge in the management of mental healthcare users (MHCUs), despite it being regarded as crucial for better health outcomes. Nurses at primary healthcare (PHC) facilities can play an important role through nursing presence in enhancing MHCUs’ medication adherence.

**Aim:**

This article aimed to investigate the relationship between MHCUs’ self-reported medication adherence and their perception of the nursing presence by registered nurses in PHC.

**Setting:**

An urban health district in Gauteng province, South Africa.

**Methods:**

A quantitative, descriptive correlational, cross-sectional design was used. The sample included 180 MHCUs. Data were collected using the Medication Adherence Rating Scale and the Presence of Nursing Scale.

**Results:**

The overall adherence level of respondents was partially adherent, with an average score of 6.45 out of a total score of 10. Respondents also reported a low level of perceived nursing presence demonstrated by registered nurses, with an average score of 72.2 out of 125. The results indicated a positive correlation between respondents’ self-reported medication adherence and their perceived nursing presence of registered nurses as evidenced by the positive value of the correlation coefficient of 0.69 with a corresponding significance probability value of 0.000 (*r* = 0.69; *p* = 0.00).

**Conclusion:**

The level of perceived nursing presence demonstrated by registered nurses played a significant role in influencing MHCUs’ level of medication adherence. The registered nurses can improve MHCUs’ medication adherence by demonstrating nursing presence skills such as good listening skills and taking care of MHCUs as individuals and not as a disease.

**Contribution:**

The results of this study confirm that there is a correlation between nursing presence and medication adherence. This holds significant value for future research in nursing presence. These findings also provide registered nurses in PHC with a valuable tool to improve medication adherence, namely nursing presence.

## Introduction

Medication adherence is an essential factor in improving the health of healthcare users and in the management of physical and mental disorders (Patton et al. 2018:138). This stance on medication adherence is referred to by Amado et al. (2015:233) as the ‘next frontier in quality improvement’. However, medication adherence remains a global challenge (Hartung et al. 2017:101). This challenge is a concern especially for mental healthcare users (MHCUs) because medication adherence is the core of their treatment (Hartung et al. 2017:101). Medication adherence leads to the prevention of relapse, improved management of the disorder and a better quality of life (Çetin & Aylaz 2018:378). It is clear from the literature that preserving MHCUs’ medication adherence is a vital but complex component of their care (Semahegn et al. 2018:10). Many studies have investigated medication adherence in an attempt to inform effective interventions to enhance adherence (Archiopoli et al. 2016:1607). One such study by Gebeyehu et al. (2019:102) highlighted that enhancing medication adherence should address numerous factors. The factors are categorised by Tham et al. (2018:537) as follows: patient-related factors, medication-related factors, healthcare providers and the healthcare system such as primary healthcare (PHC).

In the South African health system, PHC level is the first point of call for healthcare service users in the health system (Nunu & Munyewende 2017:a1262). According to a study by Murphy et al. (2015:304), the services at PHC are nurse-driven and it is of note that registered nurses (RNs) have been of importance in the provision of healthcare at this level for the past 50 years (Smolowitz et al. 2014:1). One of the services that RNs as healthcare providers at PHC are expected to provide is mental healthcare services as recommended by the World Health Organization (WHO 2008:17). It is therefore crucial to explore and enhance RNs’ skills and knowledge regarding caring for MHCUs in PHC (Jenkins et al. 2013:372).

Indeed, interventions focusing on healthcare provider factors, such as nursing presence, to improve medication adherence in MHCUs are seen as essential and should be explored (Holtzman, Brady & Yehia 2015:817). Nursing presence encompasses intentional caring behaviours by RNs; it is a present, connected and attuned *way* of providing healthcare that leads to personalised, comprehensive care that focuses on the here-and-now and is responsive to the patient’s needs (Caldeira & Timmins 2015:2356; La Cava Osterman, Schwartz-Barcott & Asselin 2010:198; Mohammadipour et al. 2017:4322; Robinson 2014:44). Nursing presence is thus a continuous practice through which MHCUs’ well-being might be promoted (Du Plessis 2016:380). Gelogahi et al. (2018:298) further stated that the resultant well-being can be physical, social, mental or spiritual well-being. Yesilot and Oz (2016:443) believed the mental well-being objective for RNs practising nursing presence is to intervene in order to change the course of the MHCUs’ health and enhancing their medication adherence would be one positive course for their health.

### Problem statement

Medication adherence contributes to the quality of life of the MHCUs and it remains the most effectual way to reach positive mental healthcare outcomes. The influence of healthcare practitioners such as RNs has been identified as one of the factors that can improve medication adherence in MHCUs. More research is needed on this factor. Holtzman et al. (2015:817) confirmed this need and recommend that research is carried out on how the characteristics of healthcare practitioners could improve medication adherence. One such characteristic, or practice, is nursing presence. Nursing presence has the potential to improve the medication adherence of MHCUs.

However, at the time of the study, there was no evidence on how the nursing presence of RNs relates to MHCUs’ medication adherence at PHC level. A literature search confirmed this knowledge gap. The literature search included searches of the databases in the North-West University’s library to gather information regarding nursing presence, medication adherence and mental healthcare services at PHCs. The search engines and databases included the following: Elton B. Stephens Company Host (EBSCOHost), ScienceDirect and Google Scholar Search. In light of the need for such research – so that appropriate interventions can be recommended and developed in future – the researchers conducted research to determine the level of MHCUs’ medication adherence, the nursing presence of RNs and to explain the relationship between these two variables. In this research, the researchers acknowledged that the level of medication adherence is self-reported by MHCUs and should be interpreted as such.

### Research questions

The main research questions in this study were as follows:

- What is the level of self-reported medication adherence of MHCUs at PHC clinics within an urban health district in Gauteng province, South Africa?
- To what extent do MHCUs perceive nursing presence from RNs at PHC clinics within an urban health district in Gauteng province, South Africa?
- What is the relationship between the self-reported medication adherence of MHCUs and their perception of the nursing presence of RNs at PHC clinics in an urban health district in the Gauteng province, South Africa?

### Hypothesis

In this study, the following hypothesis was tested:

**H1:** There is a relationship between MHCUs’ self-reported medication adherence and their perception of the nursing presence of RNs working in PHC clinics in an urban health district in the Gauteng province.

### Aim and objectives

This research study aimed to determine the relationship between MHCUs’ level of self-reported medication adherence and their perceived level of nursing presence by RNs at PHC clinics in an urban health district in Gauteng province, South Africa.

### Research objectives

The study objectives were to:

- Determine the MHCUs’ level of self-reported medication adherence at PHC clinics in an urban health district in Gauteng province, South Africa.
- Determine the MHCUs’ perceived level of the nursing presence by RNs at PHC clinics in an urban health district in the Gauteng province of South Africa.
- Determine the relationship between MHCUs’ self-reported medication adherence and their perception of the nursing presence by RNs at PHC clinics in an urban health district in Gauteng province, South Africa.

### Theoretical framework

Kostovich’s (2012:167–175) view on nursing presence guided the research. In this view, nursing presence exists as a multidimensional whole, with cognitive, affective, behavioural and spiritual experiential domains that exist in a unified, fluid manner. The view allows a nurse to bring his or her own professional knowledge and humanistic approach to care, all at the same time, without having to choose. The theory also allows a nurse to be vulnerable in their patient–nurse relationship. Both the nurse and the patient meet each other as equals within their relationship, where none is superior to the other.

Furthermore, the researchers agreed with the description of medication adherence as provided by Thompson, Kulkarni and Sergejew (2000:241–247). They highlight the self-reporting component of their definition as being a cost-effective and time-efficient measurement of medication adherence. Thompson et al. (2000:241–247) further highlighted that measuring medication adherence should also be an opportunity to address challenges hindering adherence, by indicating problematic behaviours to address them.

### Definitions of key concepts

*Mental healthcare user* is someone who receives treatment and care as prescribed by the *Mental Health Care Act* (MHCA) (SA 2002:10). In this study, the focus was on MHCUs at an urban PHC clinic.

*Primary healthcare* is defined by the WHO (2008) as:

[*T*]he first level of contact of individuals, the family and community with the national health system bringing healthcare as close as possible to where people live and work, and constitutes the first element of a continuing health care process. (p. 10)

Primary healthcare in this study meant clinics in an urban health district providing services to MHCUs.

*Nursing presence* is defined by Kostovich (2012:169) as an ‘intersubjective human connectedness shared between nurse and patient’. Nursing presence is initiated when the nurse and the patient, as vulnerable humans, enter the relationship. As trust is built, the nurse and patient risk openness and engage in the relationship. The nurse practises nursing presence as a compassionate and committed caregiver. He or she will display characteristics such as patient teaching, concern, empathy, responsive listening, coordination of care, spiritual enhancement, reassurance and personalisation of care. Nursing presence in this study meant a holistic, compassionate and committed care, which was measurable utilising scoring-displayed attributes, provided by RNs to MHCUs at PHC within an urban health district.

*Medication adherence* is defined as patients’ self-reported (a situation where respondents report their own behaviours) medication-taking behaviour, using a rating of between 0 and 10, thus grouping them as being adherent, partially adherent or non-adherent to their medication (Thompson et al. 2000:242). Medication adherence in this study meant MHCUs’ self-reported medication taking behaviours rating out of a total score of 10 at the PHC level within an urban health district.

*Self-report* is defined by Lucas (2018:1) as a measure used by researchers who wish to capture a respondent’s own evaluation or subjective experience of behaviours relating to his or her life. For this study, self-report refers to MHCUs at PHC within an urban health district report on their medication taking behaviours using a self-administered questionnaire.

A *registered nurse* is a qualified, competent person who is registered with the South African Nursing Council (SANC), who independently practicss comprehensive nursing as prescribed: capable, responsible and accountable for such practices (SA 2005).

## Research methods and design

### Study design

A quantitative, descriptive correlational and cross-sectional design was used to determine the relationship between the two study variables (Moule, Aveyard & Goodman 2017:159), namely, the medication adherence and nursing presence.

### Setting

The study setting was an urban health district in the Gauteng province of South Africa. There are 47 PHC clinics in this health district. Only 15 of these PHC clinics provide primary mental health services. This setting was sampled as it is an exemplary district in terms of integrating mental health services in PHC (DoH 2010:23) and was therefore selected. The 15 PHC clinics providing primary mental health services were all invited to participate, of which all 15 accepted the invitation.

### Study population and sampling strategy

All MHCUs in PHC in the specific urban health district in the Gauteng province who met the eligibility criteria were considered as the study population. An all-inclusive sample was used for MHCUs in PHC in an urban health district. The size of the sample was determined by the number of MHCUs at the PHC clinics during the time of data collection. The first author identified mediators at PHC clinics. The mediators recruited respondents through word of mouth and recruitment pamphlets. As a protective measure, potential participants were screened for capacity to consent by independent persons using the MacArthur Competence Assessment Tool for Clinical Research (MacCAT-CR) tool (Appelbaum & Grisso 2001:88). A total of 194 potential participants were screened for capacity to consent. Of the 194 MHCUs, only 180 MHCUs were found to have the capacity to consent to participate in the research study. Informed consent was obtained from them, using informed consent forms. Finally, 180 MHCUs formed the sample, all of whom were diagnosed with a psychiatric disorder. All respondents were receiving prescribed medication. Part C of the data collection instrument was only completed by 179 respondents.

Inclusion criteria suggested that MHCUs participating in this study had to be:

- Attending any of the 15 identified PHC clinics within the urban health district, for at least 3 months.
- Deemed capable to give consent for participation by obtaining a score of five or more on the MacCAT-CR tool.

### Data collection

After obtaining ethical clearance and the necessary permission to conduct the study, data were collected using an English self-administered questionnaire, which means that the respondents completed the questionnaires themselves (Polit & Beck 2013:414). The questionnaire was compiled using already existing questionnaires to measure the two study variables (MHCUs medication adherence and nursing presence of RNs). Permission to use the questionnaires (Medication Adherence Rating Scale [MARS] and Presence of Nursing Scale [PONS]) was granted by Professor Kulkarni for the MARS and Dr C.T. Kostovich for the PONS. The questionnaire had four sections: (1) demographic data of respondents, (2) A 26 close-ended items, five-point Likert PONS ranging from 1 (never) to 5 (always) to determine MHCUs’ perceived nursing presence by RNs at PHC (Kostovich 2012:167), (3) a 10-item, two-point MARS with either ‘yes’ or ‘no’ response to determine self-reported medication adherence of MHCUs (Thompson et al. 2000:241–247) and (4) comments respondents could have. One healthcare worker per clinic was identified and requested that they assist with the study as mediators. The first author orientated and trained the 15 mediators in the distribution and collection of the questionnaires. She delivered the questionnaires to the various clinics on the respective days of data collection, namely, one full day per clinic.

The respondents were assisted by the first author or the mediator to individually and separately complete the questionnaire. The respondents were given at least 1 hour to complete the questionnaire. Data collection was limited to a period of 1 month (August 2019) as a control measure, ensuring that respondents took part only once, namely, during their monthly visit.

Respondents were requested to participate anonymously, that is, to not indicate their names or any identifying information on the questionnaire.

The same data collection methodology was followed at each clinic. The first author kept the data safe until all data were collected. A total of 180 questionnaires were completed and were put in one box labelled ‘completed questionnaires’, well-sealed. The first author personally took the questionnaires to the statistician for data analysis.

### Data analysis

Data analysis was carried out via statistical analysis using the Statistical Package for the Social Sciences (SPSS) version 25.0 (2017) software. The assistance of the statistical consultation services of North-West University (Potchefstroom Campus) was obtained. Descriptive statistics were obtained, including frequency distribution, percentages, mean and standard deviation (s.d.). Furthermore, correlational statistics were determined by calculating correlational coefficients (*r*), *t*-tests and Analysis of variance (ANOVA).

### Reliability and validity

#### Reliability

Cronbach’s alpha coefficient was used to calculate the reliability of the data collection instruments. A score of 0.8 and higher was seen as acceptable in this study (Grove, Burns & Gray 2013:391).

In previous studies the MARS instrument was tested on MHCUs and the Cronbach’s alpha coefficient was 0.75 (Thompson et al. 2000:242) and 0.76, respectively (Owie, Olotu & James 2018:85), which indicates good reliability. The Cronbach’s alpha coefficient was 0.87 in this research, which further confirmed the reliability of the instruments used.

Regarding the PONS, the Cronbach’s alpha coefficient measured 0.95, which also indicated reliability (Kostovich 2012:171–172). In this study, the Cronbach’s alpha coefficient of the PONS was 0.98 and could therefore be accepted as a reliable instrument to measure nursing presence.

#### Validity

The MARS and PONS were presented to the statistician before the commencement of the study, who confirmed that the questions were clear and unambiguous. The questions were therefore not adapted for this study. In addition, to determine if the questions were clear (Botma et al. 2010:275), understandable and easy to complete, the questionnaire was pre-tested with five MHCUs. Face validity was thus ensured.

### Ethical considerations

Ethical clearance to conduct the study was obtained from the Ethics Committee of the North-West University (Certificate No.: NWU-0015-08-S1). Thereafter, approval was obtained from the Gauteng Department of Health and the Health District in which the study was conducted, with approval reference (REF: NRHD GP 201810_061). Primary healthcare clinics’ managers then gave goodwill permission in writing. Throughout the study, the principles of beneficence, non-maleficence, equality and respect for persons were adhered to (Department of Health of the Republic of South Africa 2015:14–17), as explained throughout the discussion of the research method. The researcher maintained objectivity throughout the study and ensured the ongoing safety of the respondents by confirming that the study did not expose them to any sort of harm.

## Results

The demographic profile of the respondents is discussed. The results regarding the self-reported medication adherence of MHCUs are then presented, followed by the results regarding the respondents’ perceived nursing presence by RNs. Then, the relationship between the respondents’ self-reported medication adherence and their perceived nursing presence by RNs is discussed. Finally, the respondents’ comments are summarised.

### Demographic and general information

An adequate sample of 180 respondents, based on a Kaiser-Meyer-Olkin test (KMO) value of 0.857 (Weis & Schank 2017:405), participated. Table 1 shows that out of 180 respondents, there was an almost equal distribution of gender. Firstly, the age of the respondents clustered around 30–40 years of age, constituting a quarter (*n* = 45; 25%) of the entire sample. More than half (*n* = 95; 55%) of the sample only reached high school as their highest educational level. The majority of the sample was African (*n* = 139; 77.2%) MHCUs, followed by white (*n* = 24; 13.3%), mixed race (*n* = 12; 6.7%) and other races (*n* = 5; 2.8%). A total of 119 (66.1%) respondents were single, while a total of 49 (23.2%) respondents indicated that they were married.

**TABLE 1:** Demographic information of participants (*n* = 180).

Demographics	Variables	Frequency	Percent (%)
Gender	Male	91	50.80
	Female	88	49.20
Age (in years)	Below 30	30	16.70
	30–40	45	25.00
	41–50	38	21.10
	51–60	43	23.90
	Above 60	24	13.30
Highest level of education	Primary school	61	33.90
	High school	99	55.00
	College	17	9.40
	University	3	1.70
Race	Black	139	77.20
	White	24	13.30
	Mixed race	12	6.70
	Other	5	2.80
Marital status	Single	119	66.10
	Married	49	27.20
	Divorced	12	6.70

*Source*: Kalimashe, L., 2019, *The relationship between mental health care users medication adherence and the nursing presence of registered nurses in primary health care*, Mini-dissertation for Masters degree, North-West University, Potchefstroom

### Description of study variables

#### Level of self-reported medication adherence of the study sample

The MARS was only completed by 179 respondents (one respondent did not complete the MARS). The respondents’ responses per item of the MARS questionnaire are presented in Table 2. Responses are presented descriptively, namely, through frequency counts, percentages, means and s.d. Four areas of concern were identified (items 4, 6, 7 and 8). These four items are presented in the order of strength. As much as 74.9% of the 179 respondents close to three-quarters of the 179 MHCUs reported that adhering to taking their medication did not prevent them from getting sick (74.9%). Secondly, 130 (72.6%) respondents indicated that their thoughts are not clear while on medication. Another concern was that as many as 118 (65.9%) respondents felt that it was unnatural for their mind and body to be controlled by medication. Also, 100 (55.9%) of the 179 respondents agreed that they stopped taking medication when they felt worse while taking it.

**TABLE 2:** Responses of mental healthcare users on medication adherence rating scale.

Item	Question	Yes		No		Mean	Standard deviation
		*N*	%	*N*	%		
1	Do you ever forget to take your medication?	41	22.90	138	77.10	0.77	0.421
2	Are you careless at times about taking your medication?	71	39.70	108	60.30	0.6	0.491
3	When you feel better, do you sometimes stop taking your medication?	66	36.90	113	63.10	0.63	0.484
4	Sometimes if you feel worse when you take the medication, do you stop taking it?	100	55.90	79	44.10	0.44	0.498
5	I take my medication only when I am sick.	25	14.00	154	86.00	0.86	0.348
6	It is unnatural for my mind and body to be controlled by medication.	118	65.90	61	34.10	0.34	0.475
7	My thoughts are clearer on medication (Reversed).	49	27.40	130	72.60	0.73	0.447
8	By staying on medication, I can prevent getting sick (Reversed).	45	25.10	134	74.90	0.75	0.435
9	I feel weird, like a ‘zombie’ on medication.	41	22.90	138	77.10	0.77	0.421
10	Medication makes me feel tired and sluggish.	80	44.70	99	55.30	0.55	0.499

*Source*: Kalimashe, L., 2019, *The relationship between mental health care users medication adherence and the nursing presence of registered nurses in primary health care*, Mini-dissertation for Masters degree, North-West University, Potchefstroom

Overall mean = 0.85 and standard deviation = 0.49.

However, there were also positive findings, namely, that as many as 156 (86.0%) respondents indicated that they continued to take their medication not only when they were ill, 138 (77.1%) reported that they did not forget to take their medication, 113 (63.1%) continued to take their medication even if they were feeling better and 108 (60.3%) reported that they were not careless about taking their medication. The majority of the respondents also reported adherence regarding items 9 and 10 of the MARS.

**FIGURE 1:**
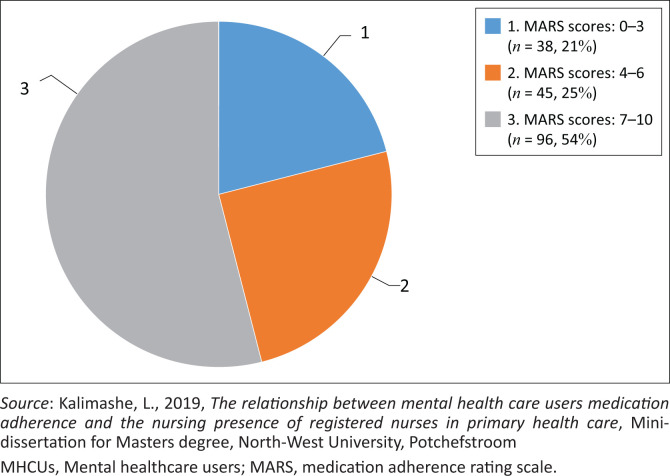
Mental healthcare users’ level of medication adherence.

The overall level of self-reported medication adherence is presented in Figure 1, where the frequency distribution of the respondents’ total MARS scores is shown. The total scores on the MARS could range between 0 and 10, where a higher score indicates higher medication adherence (Owie et al. 2018:87). The sum of scores for all item scores was calculated for each respondent to determine their level of self-reported medication adherence. These scores were interpreted as non-adherence (a score range of 0–3), partial adherence (4–6) and good adherence to medication (7–10) (Johnson et al. 2016:699). The findings of this research show that 96 (54%) respondents reported good adherence to their medication, 45 (25%) reported partial adherence and 31 (21%) of respondents reported non-adherence (see Figure 1). The mean MARS score for all respondents indicates partial adherence by the sample, namely, a score of 6.45.

#### Level of nursing presence of registered nurses

**FIGURE 2:**
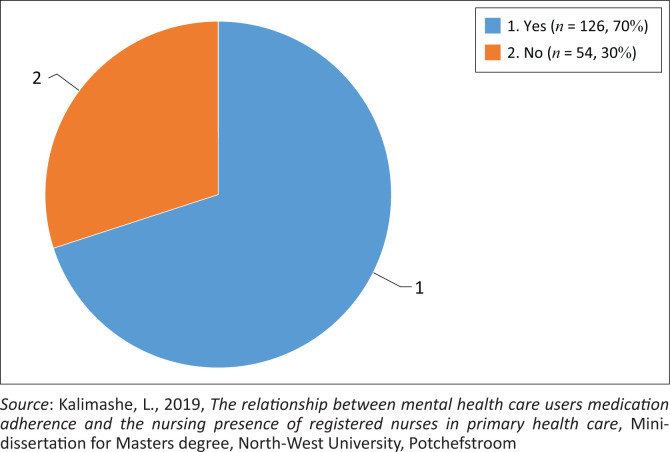
Perception of mental healthcare users on the existence of nursing presence (Presence of these registered nurses made a difference in your life).

Firstly, it was determined whether respondents were of the opinion that RNs at PHC clinics practised nursing presence and whether nursing presence made a difference in their lives (the first question on the PONS). Figure 2 shows this feedback. Nearly one-third of the respondents (*n* = 54; 30%) perceived that RNs did not practise nursing presence, and the remaining 126 (70%) respondents agreed that RNs practised nursing presence. These 126 respondents were requested to answer items 2–26 of the PONS in order to ascertain their views on the levels of nursing presence practised by RNs.

Table 3 summarises the different attributes of nursing presence, as represented in items 2–26 of the PONS instrument. This summary shows the respondents’ views on how often the RNs demonstrated these attributes, as indicated by frequency counts, percentages, mean and s.d.

**TABLE 3:** Responses of mental healthcare users on the presence of nursing scale.

Item	These registered nurses	Never		Rarely		Occasionally		Frequently		Always		Mean	Standard deviation
		*N*	%	*N*	%	*N*	%	*N*	%	*N*	%		
2	Were open to my concerns.	1	0.80	21	16.70	63	50.00	41	32.50	0	0.00	3.1	0.7
3	Taught me what I needed to know.	2	1.60	22	17.50	68	54.00	34	27.00	0	0.00	3.1	0.7
4	‘Checked’ on me.	4	3.20	65	51.60	43	34.10	14	11.10	0	0.00	2.5	0.7
5	Met my spiritual needs.	57	45.60	63	50.40	2	1.60	3	2.40	0	0.00	1.6	0.6
6	Talked to me as a friend.	1	0.80	22	17.50	73	57.90	30	23.80	0	0.00	3.0	0.7
7	Physically comforted me.	1	0.80	40	31.70	64	50.80	21	16.70	0	0.00	2.8	0.7
8	Emotionally comforted me.	1	0.80	11	8.70	67	53.20	47	37.30	0	0.00	3.3	0.7
9	Understood my feelings.	1	0.80	35	27.80	69	54.80	20	15.90	1	0.80	2.9	0.7
10	Earned my trust.	1	0.80	21	16.70	60	47.60	43	34.10	1	0.80	3.2	0.7
11	Were skilled in providing my care.	1	0.80	10	7.90	60	47.60	49	38.90	6	4.80	34	0.7
12	Were there if I needed them.	3	2.40	49	38.90	59	46.80	14	11.10	1	0.80	2.7	0.7
13	Helped my day run smoothly.	1	0.80	24	19.00	75	59.50	26	20.60	0	0.00	3.0	0.7
14	Created a sense of healing around me.	1	0.80	16	12.70	75	5.50	31	24.60	3	2.40	3.2	0.7
15	Listened and responded to my needs.	3	2.40	33	26.20	73	57.90	17	13.50	0	0.00	2.8	0.7
16	Calmed my fears.	2	1.60	32	25.40	67	53.20	24	19.00	1	0.80	2.9	0.7
17	Were concerned about me.	3	2.40	43	34.10	60	47.60	20	15.90	0	0.00	2.8	0.7
18	Were committed to care for me.	1	0.80	14	11.10	74	58.70	36	28.60	1	0.80	3.2	0.7
19	Made me feel safe.	2	1.60	18	14.30	72	57.10	32	25.40	2	1.60	3.1	0.7
20	Made me feel at peace.	2	1.60	26	20.60	65	51.60	31	24.60	2	1.60	3.0	0.8
21	Took care of me as a person, not as a disease.	4	3.20	25	19.80	48	38.10	35	27.80	14	11.10	3.2	1.0
22	Gave me as much control over my healthcare as possible.	36	28.60	83	65.90	5	4.00	2	1.60	0	0.00	1.8	0.6
23	Made the quality of my life better.	2	1.60	19	15.10	72	57.10	31	24.60	2	1.60	3.1	0.7
24	I had confidence in these.	1	0.80	18	14.30	67	53.20	33	26.20	7	5.60	3.2	0.8
25	Felt a connection between myself and them.	10	7.90	42	33.30	56	44.40	18	14.30	0	0.00	2.7	0.8
26	Their presence made a difference to me.	4	3.20	19	15.10	63	50.00	38	30.20	2	1.60	3.1	0.8

*Source*: Kalimashe, L., 2019, *The relationship between mental health care users medication adherence and the nursing presence of registered nurses in primary health care*, Mini-dissertation for Masters degree, North-West University, Potchefstroom

Overall mean = 2.91 and standard deviation = 0.72.

Respondents mostly indicated that RNs practised the attributes of nursing presence ‘occasionally’, ‘rarely’ or ‘frequently’, with their most frequent response being ‘occasionally’. Very few participants chose the extreme options of ‘never’ or ‘always’. This leads to an overall mean score of 2.9, which translates to 3 on a five-point Likert scale.

However, regarding the items ‘caring for their spiritual needs’ and ‘giving respondents control over their healthcare as much as possible’, respondents mostly indicated ‘never’ or ‘rarely’. ‘Never’ was chosen by 57 (45.6%) and 36 (28.6%) respondents for these two items, respectively, while ‘rarely’ was chosen by 63 (50.4%) and 83 (65.9%) respondents, respectively. These two items had the lowest mean values (1.6 ± 0.6 and 1.8 ± 0.6) of all the items on the PONS, well below the overall mean score of 2.9 ± 0.72.

Furthermore, the responses of the respondents indicated that they experienced RNs as being open to their concerns, teaching them what they needed to know, talking to them as friends to foster therapeutic communication for a therapeutic relationship, being emotionally comforting and understanding their feelings, although these attributes were only marked as being occasionally demonstrated by RNs. Registered nurses were experienced as being generally helpful in enabling the respondents’ days to run smoothly, although this was also only perceived to happen on an occasional basis. The respondents also reported that RNs occasionally create a sense of healing, thus contributing to making a difference in their lives.

Respondents perceived that the RNs have fairly good listening skills, as 73 (57.9%) of them reported that RNs listened and responded to their needs. In addition, just over 50% (*n* = 67; 53.2%) of the respondents felt that the RNs occasionally demonstrated the ability and readiness to calm their fears. Registered nurses were perceived to occasionally show commitment and made respondents feel safe and at peace, improving the quality of respondents’ lives. Just over half (*n* = 67; 53.2%) of the respondents had confidence in the RNs and RNs were perceived to have occasionally made a difference in their lives. In all the above-mentioned items, more than 50% of the respondents chose ‘occasionally’ as a response.

Some respondents, however, perceived that the above attributes were displayed ‘frequently’ by the RNs. Notable among these items were the following: RNs being open to the concerns of respondents (*n* = 41; 32.5%), teaching them what they needed to know (*n* = 34; 27%), being emotionally comforting (*n* = 47; 37.3%) and earning their trust (*n* = 43; 34.1%). In addition, RNs were perceived to frequently exhibit skills in providing care to the respondents and showing commitment (*n* = 36; 28.6%), including taking care of respondents as individuals and not as a disease (*n* = 35; 27.8%) and their presence making a frequent difference to 38 (30.2%) of the 126 respondents.

Table 4 indicates the total scores per respondent. It is clear that 75 respondents (59.5%) perceived RNs at PHCs to practise low levels of nursing presence (score ranges 25–50 and 51–75). The remaining 51 respondents (40.5%) perceived RNs at PHCs to practise high levels of nursing presence (score ranges 76–100 and 101–125). The mean PONS score was 72.2 ± 15.3 on the 25–125 scale.

### Relationship between mental healthcare users’ medication adherence and the nursing presence of registered nurses

Whether the index for the MARS had a relationship with the index on the PONS was established through a Pearson’s correlation coefficient. The findings revealed a correlation coefficient for the two parameters of 0.69, which was statistically significant (0.000 < 0.01) because the *p*-value (0.000) was less than 0.01. Thsus, a statistically significant correlation exists.

### Comments made by mental healthcare users

Out of 180 completed questionnaires, only 42 respondents used the comments section (section D of the questionnaire) by writing down anything they wanted to share. The comments from respondents were about the RNs, the clinic, respondents themselves and their medication and confirm the statistical results. Examples of the comments are:

- Nurses are sometimes nice to me but I still feel they don’t understand my illness.
- The nurses say they can’t change my medication when I complain, it can only be done by a doctor but I last saw a doctor a year ago.
- My complaints about medication side effects are not being addressed.
- I take less of what I’m told to take because the medication makes me too tired.
- I’m pleased with my medication.
- I still got admitted even though I was taking my medication as told.

## Discussion

The results of this study revealed that self-reported medication adherence and the perception of whether RNs display presence were not significantly influenced by some of the demographic characteristics. The gender of respondents did not have any influence on their self-reported medication adherence or their perceived level of nursing presence by RNs. Similar findings were seen by Kostovich (2012:172) regarding the PONS and by Owie et al. (2018:88) regarding MARS scores across gender (*t* = 0.25, *p* = 0.80). Furthermore, respondents’ marital status did not affect the level of perceived nursing presence. However, regarding medication adherence, there was a small practical significance (*d*) of 0.20 noted for married respondents compared with single respondents. Tham et al. (2018:805) noted similar findings.

A large practical significance regarding medication adherence was observed in terms of respondents’ race. Evidence was found which suggests that medication adherence is significantly dependent (*p* = 0.017) on the race of the respondents. A large practical significance was noticed between African and mixed race respondents (*d* = 0.85) and between white and mixed race respondents (*d* = 0.81). Regarding the perceived nursing presence by RNs, only a medium practical significance between African and mixed race respondents (*d* = 0.73) and between white and mixed race (*d* = 0.65) respondents was observed.

Regarding self-reported medication adherence overall, it was apparent that respondents reported partial adherence. As many as 83 (46%) respondents scored below 7 and were thus either non-adherent or partially adherent to their medication. Chaudhari et al. (2017:215) similarly reported low adherence to medication by MHCUs, namely, that only 48% of their sample were adherent to taking their medication. In our study, we concluded that non- or partial-adherence could be related to respondents’ behaviour to stop taking their medication when it was making them feel worse and to their beliefs that it was unnatural for their minds and bodies to be controlled by medication and that staying on medication can prevent them from getting sick or give them clearer thoughts.

Regarding the PONS, the respondents perceived RNs to demonstrate low levels of nursing presence. The mean PONS score of 72.2 ± 15.3 on the 25–125 scale is lower than the mean score of 89.05 ± 22.50 found by Yesilot and Oz (2016:447). In our study, it is concerning that more than 90% of respondents perceived that RNs never or rarely allow them control over their healthcare or attend to their spiritual needs. Furthermore, respondents perceived that RNs only occasionally demonstrated nursing presence towards them. Respondents did perceive that RNs were skilled in providing care (mean score of 3.4). From this discussion, it is clear that there is a need for strengthening the practice of nursing presence by RNs.

Of specific importance is the finding that there is a correlation between self-reported medication adherence and nursing presence, as confirmed by a Pearson’s correlation coefficient of 0.69, which indicates a positive relationship between the two study variables (medication adherence and nursing presence). This positive correlation implies that MHCUs who perceive RNs as displaying higher levels of nursing presence are most likely to report high levels of medication adherence. This finding strongly supports the argument that nursing presence can be a strategy to improve medication adherence in MHCUs.

### Strengths and limitations

This research fills a knowledge gap, namely the question of whether there is a relationship between MHCUs’ self-reported medication adherence and the perceived nursing presence of RNs. The findings of this research have the potential to enrich existing knowledge regarding medication adherence (MARS scale) and nursing presence (PONS scale).

Looking at limitations, it is acknowledged that this study depended on self-reporting and that it will be difficult to generalise the results because of the small sample size and the sampling method (all-inclusive sample). It is acknowledged that an objective assessment, the inclusion of rural areas, nurses’ perception and the perceptions of other members of the multidisciplinary team could have added value.

### Recommendations

#### Recommendations for nursing practice

Primary healthcare clinics should consider prioritising a monitoring system to monitor MHCUs’ medication adherence. A collaborative approach should be considered by RNs in working collaboratively with MHCUs to enhance their medication adherence. A nursing presence approach should be considered as an effective way to enhance medication adherence. Practices such as giving MHCUs as much control over their healthcare as possible by discussing treatment options with them should be considered and listening to them and addressing their concerns regarding medication side effects and equipping them with the knowledge to improve insight.

#### Recommendations for future research

Further research should be conducted to explore nursing presence and its value and effect on healthcare service users. This should include not only qualitative research but also action research to encourage nurses to practise nursing presence. Research on the factors and characteristics inherent to healthcare practitioners that enhance MHCUs’ medication adherence will also be meaningful.

## Conclusion

The hypothesis of the research was supported as the relationship between the study variables was confirmed and the aim of the study was achieved. The nursing presence of RNs indeed plays a role in MHCUs’ medication adherence. If nursing presence is encouraged and promoted among RNs in PHC, MHCUs will have improved medication adherence, resulting in positive health outcomes.
